# No significant effect on bone mineral density by high doses of vitamin D_3 _given to overweight subjects for one year

**DOI:** 10.1186/1475-2891-9-1

**Published:** 2010-01-07

**Authors:** Rolf Jorde, Monica Sneve, Peter A Torjesen, Yngve Figenschau, John-Bjarne Hansen, Guri Grimnes

**Affiliations:** 1 Institute of Clinical Medicine, University of Tromsø, and Medical Clinic, University Hospital of North Norway, Tromsø, Norway; 2 Department of Ophthalmology and Neurosurgery, Division of Ophthalmology, University Hospital of North Norway, Tromsø, Norway; 3 Hormone Laboratory, Aker University Hospital, Oslo, Norway; 4 Department of Medical Biochemistry, University Hospital of North Norway, and Institute of Medical Biology, University of Tromsø, Tromsø, Norway; 5 Center for Atherothrombotic Research in Tromsø (CART), Institute of Clinical Medicine, University of Tromsø, Norway

## Abstract

**Background:**

In meta-analyses supplementation with vitamin D appears to reduce incidence of fractures, and in cross-sectional studies there is a positive association between serum 25-hydroxyvitamin D (25(OH)D) levels and bone mineral density (BMD). However, the effect of supplementation with high doses of vitamin D on BMD is more uncertain and could in theory have both positive and negative effects.

**Methods:**

The study was a one year, double blind placebo-controlled intervention trial performed at the University Hospital of North Norway. 421 subjects, 21 - 70 years old, were included and 312 completed the study. The subjects were randomized to vitamin D_3 _40.000 IU per week (DD group), vitamin D_3 _20.000 IU per week (DP group), or placebo (PP group). All subjects were given 500 mg calcium daily. Serum 25(OH)D, osteoprotegrin (OPG), receptoractivator of nuclear factor-kappaB ligand (RANKL), and BMD at the lumbar spine and the hip were measured before and at the end of the study.

**Results:**

At baseline the mean serum 25(OH)D levels were 58 nmol/L (all subjects) and increased to 141 and 100 nmol/L in the DD and DP groups, respectively. After one year, no significant differences were found between the three groups regarding change in BMD, serum OPG or RANKL.

**Conclusions:**

Supplementation with high doses of vitamin D for one year does not appear to have a negative effect on BMD in healthy subjects. In order to disclose a positive effect, subjects with low BMD and/or low serum 25(OH)D levels need to be studied.

**Trial registration:**

The trial was registered at ClinicalTrials.gov (NCT00243256).

## Background

A sufficient intake of vitamin D, or sun exposure for production of vitamin D in the skin, is of vital importance for skeletal health, and patients with severe vitamin D deficiency exhibit hypocalcemia and rickets or osteomalacia [1].

The skeleton is constantly remodelled. This occurs in small bone remodelling units where the balance between bone resorption by the osteoclasts and bone matrix synthesis by the osteoblasts determines the volume and quality of the bone [2]. It has been known for many years that the activity of the osteoclasts is influenced by the osteoblast, which was clarified by the recent discovery of the osteoprotegrin (OPG)/receptoractivator of nuclear factor-kappaB (RANK)/RANK ligand (RANKL) system [2-6]. Thus, the osteoblasts and the bone marrow stromal cells produce RANKL which activates the osteoclasts by binding to its RANK receptor. The osteoblasts also produce OPG which functions as a decoy receptor and binds RANKL and thereby prevents it from binding to RANK [2,7].

The classical effect of 1,25-dihydoxyvitamin D (1,25(OH)_2_D), which is the active form of vitamin D, is to increase the intestinal calcium absorption and thereby have an indirect effect on bone formation [1]. In addition, 1,25(OH)_2_D has direct effects on bone, and receptors for 1,25(OH)_2_D (VDR) have been found in osteoclasts [8] as well as in osteoblasts [9]. Traditionally, 1,25(OH)_2_D has been considered a bone-resorbing hormone. This was based on its potent stimulation of bone resorption in tissue cultures [10,11] which was further substantiated by the finding that 1,25(OH)_2_D increased the RANKL expression [5,11]. However, active vitamin D metabolites have also been reported to inhibit bone resorption [12] and may therefore have a dual effect on bone formation [11].

From these in vitro experiments it is hard to predict the effect of vitamin D supplementation on bone in humans provided there is not a severe vitamin D deficiency. If the effect is positive, one would expect that supplementation with vitamin D would prevent osteoporotic fractures which was demonstrated in a recent meta-analysis by Bischoff-Ferrari et al. [13]. A dose dependency was also reported, and no effect was seen with 400 IU per day. Similarly, in a large cross-sectional study there was a positive relation between serum 25(OH)D, which is the storage form of the vitamin and the one used to evaluate a subject's vitamin D status, and bone mineral density (BMD) [14]. However, for subjects above the age of 50 years, the BMD reached a plateau at serum 25(OH)D levels of 90 - 100 nmol/L, and thereafter appeared to decline. If this reflects a causal relation, vitamin D given in high doses may have a negative effect on bone. Given the focus on the need for higher doses of vitamin D [15] this is an important issue to settle, but so far, most intervention studies have used vitamin D in doses of 800 IU per day or less [13].

We have recently performed a one year placebo-controlled intervention study with vitamin D in doses of 20.000 IU and 40.000 IU per week with change in weight as primary end point [16]. In addition, bone densitometry was performed and serum OPG and RANKL measured before and at the end of the study. This gave us the opportunity to address the issue of skeletal effects of high dose vitamin D supplementation.

## Methods

The protocol has previously been described in detail [16]. The subjects were recruited by advertisements in local newspapers and from our outpatient clinic. The subjects were initially screened at our outpatient clinic at the Department of Internal Medicine at the University Hospital of North Norway. All potential participants were told that they would be part of a weight loss study assessing the effect of high doses of vitamin D on body weight. Males and females 21 to 70 years old, with BMI between 28.0 - 47.0 kg/m^2 ^were included. Subjects with diabetes or a history of coronary infarction, angina pectoris, stroke, renal stone disease, or sarcoidosis were excluded. Subjects with serum calcium > 2.55 mmol/L, males with serum creatinine > 129 μmol/L and females with serum creatinine > 104 μmol/L, and subjects using bisphosphonates or oestrogen (for contraception or replacement) were not included.

At baseline fasting blood samples were drawn and any previous supplements with calcium and vitamin D (including cod liver oil) were discontinued. All subjects were given a daily supplement with 500 mg calcium (Nycoplus Calcium^®^, Nycomed, Norway) throughout the one year intervention period. The participants were given oral information and written recommendations on healthy diet and physical activity. The study was a randomized, double blind clinical trial. Using block-randomization, the subjects were randomized into one of three groups, stratified by gender and smoking status: one group was to take two capsules of vitamin D_3 _(20 000 IU cholecalciferol per capsule (Decristol^®^, Jenapharm, Jena, Germany)) per week; one group one capsule of vitamin D_3 _and one placebo capsule per week; and one group two placebo capsules per week. The subjects were supplied with new medication every third month. Unused calcium tablets and capsules were returned and counted. The subjects were classified as current smokers or current non-smokers. Non-fasting blood samples for serum calcium analysis were drawn after 3, 6, and 9 months to disclose development of hypercalcemia.

### Measurements

Height and weight were measured wearing light clothing and no shoes. Serum calcium and parathyroid hormone (PTH) were measured as previously described [17]. Serum levels of 25-hydroxyvitamin D (25(OH)D) were measured by radioimmunoassay (DiaSorin, Stillwater, MN, USA). This assay measures both 25(OH)D_3 _and 25(OH)D_2_, and the intra- and total assay coefficients of variation (CVs) are 6% and 14%, respectively [18]. BMD was determined by anterior-posterior dual-energy X-ray absorptiometry (DEXA) scans at the lumbar spine and hip according to the manufacturer (GE Lunar Prodigy, LUNAR Corporation, Madison, WI, USA). The mean of L2-L4 and the mean of the right and left total hip values were used in the analyses (CV 3.6%). Serum OPG was measured as previously described with an intra-assay CV of 3.2% and inter-assay CV of 11.1% [19]. The concentration of free RANKL was measured by a new, highly sensitive ELISA assay for free RANKL with a detection limit of 0.02 pmol/l, an intra-assay CV of 9.6% and an inter-assay CV of 15.3% (ampli sRANKL human, Biomedica, Vienna, Austria). The analysis was performed according to the manufacturer's instruction. The analyses of serum OPG and RANKL were performed on coded samples without knowledge of clinical status by the person performing the assays. All samples were analyzed in duplicate and the mean value is used in this report. OPG and RANKL were measured in baseline sera in all subjects who completed the study and in sera from the end of the study in a random sample of subjects in the DD and PP groups as appear in the tables.

### Statistical analyses

Normal distribution was evaluated with visual inspection of histograms with normal curve, and determination of skewness and kurtosis. All dependent variables except serum OPG and RANKL were considered normally distributed at baseline. After log transformation OPG attained normal distribution and was used as such when parametric statistics were applied. Because of several 0-values (not detectable) RANKL could not be log transformed and therefore was evaluated with non-parametric statistics. The delta values for OPG and RANKL were also not normally distributed and could not be log transformed because of several 0-values, and were therefore evaluated with non-parametric statistics. The other delta values were normally distributed. A multiple linear regression model with age, gender, BMI, smoking status, serum 25(OH)D, serum PTH and serum calcium as covariates was used to evaluate individual predictor of BMD L2-L4, BMD total hip and serum OPG. Correlations were evaluated with Pearson's or Spearman's correlation coefficients as appropriate.

The intervention study was analysed per protocol. Comparisons between the groups at baseline and between their delta values (value at end of the study minus value at baseline) were performed with ANOVA, the Kruskall Wallis test or the Chi-square test. The Bonferroni correction was used where multiple comparisons were performed. Unless otherwise stated, data are expressed as mean ± SD. All tests were done two-sided, and *P*-value < 0.05 was considered statistically significant. The Statistical Package for Social Sciences version 15.0 was used for all statistical analyses (SPSS Inc., Chicago, Ill., USA).

The power calculation was performed according to the primary end point (weight loss) to disclose a clinically significant difference of 6 kg with or without vitamin D supplementation [16].

### Ethics

The study was approved by the Regional Ethics Committee. All participants gave written informed consent prior to the study.

## Results

### Baseline

**Figure 1:**
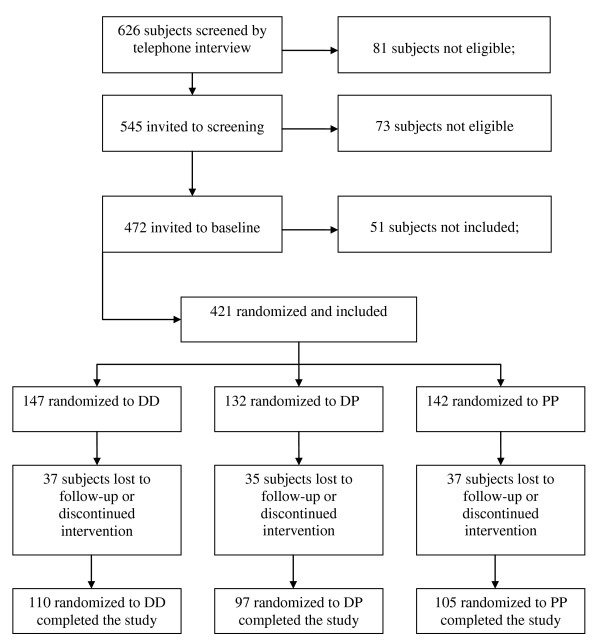
**Study consort diagram**.

**Figure 2:**
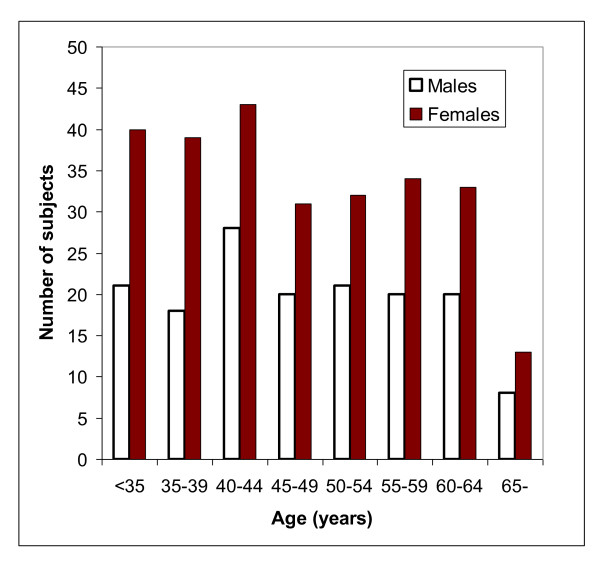
**Age distribution in the 156 males and 265 females at baseline**.

**Figure 3:**
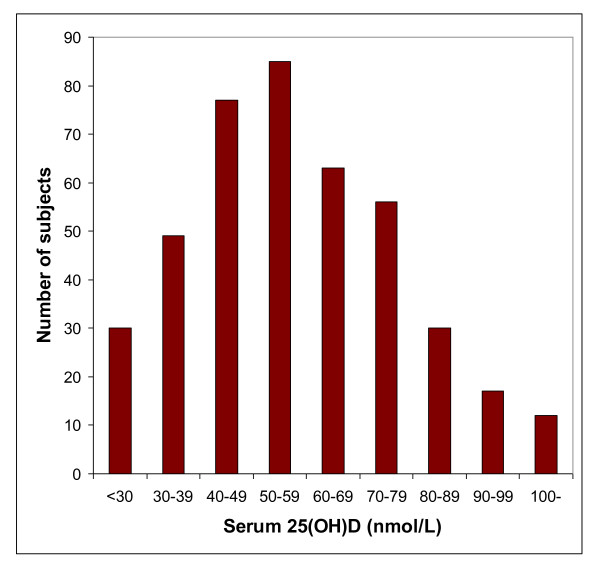
**Distribution of serum 25(OH)D in the 421 subjects at baseline**.

The inclusion period started in November 2005 and the last person was included in October 2006. Of 626 subjects initially screened by telephone interview, 421 (156 men and 265 women) met the inclusion criteria and had complete datasets (Figure 1). The baseline characteristics of these subjects are shown in Table 1. Among the women, 114 were postmenopausal. The age distribution among the men and women are shown in Figure 2. The mean serum 25(OH)D levels was 57.7 ± 20.7 nmol/L and the distribution is shown in Figure 3. As expected there were a number of significant univariate correlations between the parameters measured (Table 2). However, after adjusting for confounders in the multiple linear regression model only a few of these relations remained significant. In particular, serum PTH remained as a significant and negative predictor of BMD both for L2-L4 and the hip, and female gender and high age were associated with Lg OPG (Table 3). Serum OPG was negatively correlated with BMD L2-L4 and BMD at the hip, but not after adjusting for the other covariates (data not shown). Serum RANKL was below the detection limit in 141 of the 336 subjects with RANKL measurement at baseline, and no association was found with any of the other parameters measured.

**Table 1:** Baseline characteristics of all the 421 subjects included in the study

Males (%)	37.1
Age (years)	47.1 ± 11.4
BMI (kg/m^2^)	34.8 ± 3.9
Smokers (%)	22.3
BMD L2-L4 (g/cm^2^)	1.256 ± 0.163
BMD total hip (g/cm^2^)	1.096 ± 0.136
OPG (pg/ml)^1^	1970 ± 672
RANKL (pg/ml)^1^	0.08 ± 0.17
Serum 25(OH)D (nmol/L)	57.7 ± 20.7
Serum PTH (pmol/L)	5.41 ± 1.87
Serum calcium (mmol/L)	2.31 ± 0.11

^1^N = 336

**Table 2:** Pearson's correlation coefficient between baseline values in the 421 subjects

	Age (years)	BMI (kg/m^2^)	BMD L2-L4 (g/cm^2^)	BMD total hip (g/cm^2^)	Lg OPG (pg/ml)^1^	RANKL (pg/ml)^1^	Serum 25(OH)D (nmol/L)	Serum PTH (pmol/L)
Age (years)								
BMI (kg/m^2^)	- 0.15^†^							
BMD L2-L4 (g/cm^2^)	- 0.21^‡^	0.04						
BMD total hip (g/cm^2^)	- 0.36^‡^	0.24^‡^	0.64^‡^					
Lg OPG (pg/ml)^1^	0.38^‡^	- 0.04	- 0.13*	- 0.15^†^				
RANKL (pg/ml)^1^	- 0.06^2^	- 0.08^2^	- 0.01^2^	- 0.03^2^	- 0.02^2^			
Serum 25(OH)D (nmol/L)	0.31^‡^	- 0.16^†^	0.04	- 0.07	0.20^‡^	0.07^2^		
Serum PTH (pmol/L)	0.08	0.19^‡^	- 0.18^‡^	- 0.11*	0.01	- 0.05^2^	- 0.26^‡^	
Serum calcium (mmol/L)	0.30	0.02	0.02	0.05	0.09	- 0.08^2^	0.06	- 0.10*

*P < 0.05; ^†^*P *< 0.01; ^‡^*P *> 0.001

^1^N = 336 ^2^Spearman's correlation coefficient

**Table 3:** Baseline relations evaluated with multiple linear regression in the 421 subjects. Values are standardized β-coefficients.

Dependent variables	R^2^	Independent variables in the model						
		
								
		**Age (years)**	**BMI (kg/m^2^)**	**Gender^1^**	**Smoking status^2^**	**Serum 25(OH)D (nmol/L)**	**Serum PTH (pmol/L)**	**Serum calcium (mmol/L)**
BMD L2-L4 (g/cm^2^)	0.10	- 0.22^‡^	0.06	- 0.13^†^	0.07	0.06	- 0.17^‡^	0.01
BMD total hip (g/cm^2^)	0.25	- 0.34^‡^	0.24^‡^	- 0.25^‡^	0.05	0.03	- 0.13^†^	0.04
Lg OPG (pg/ml)^3^	0.17	0.36^‡^	0.04	0.11*	0.07	0.07	0.01	0.09

*P < 0.05; ^†^*P *< 0.01; ^‡^*P *> 0.001

^1^Males = 1, females = 2; ^2^Smokers = 1, non-smokers = 2; ^3^N = 336

### Intervention study

One hundred and forty-seven, 132 and 142 subjects were randomized to the DD, DP and PP groups, respectively. 110 subjects in the DD group, 97 subjects in the DP group, and 105 subjects in the PP group completed the study (Figure 1). The reasons for the dropouts (37 subjects in the DD group, 35 subjects in the DP group and 37 subjects in the PP group) have previously been described in detail [16]. However, most of those who dropped out withdrew their consent for participation without stating a specific reason. The compliance rate in the DD, DP and PP groups were for the vitamin D/placebo capsules 95%, 96%, 96%, and for the calcium tablets 82%, 84%, and 83%, respectively. At baseline, those in the DP group had BMI 1.5 kg/m^2 ^lower than those in the PP group (*P *< 0.05). Apart from this difference, the DD, DP and PP groups were similar (Table 4).

**Table 4:** Baseline and delta values in those who completed the study

	Baseline values			Delta values (value at end of study minus baseline)		
		
	DD group	DP group	PP group	DD group	DP group	PP group
N	110	97	105			
Males (%)	40.0	40.2	39.0			
Age (years)	47.3 ± 11.1	47.7 ± 11.6	50.8 ± 10.7			
BMI (kg/m^2^)	34.4 ± 3.9	33.7 ± 3.5*	35.2 ± 3.9	0.01 ± 1.33	0.13 ± 1.10	0.09 ± 1.35
Smokers (%)	20.9	20.6	17.1			
BMD L2-L4 (g/cm^2^)	1.270 ± 0.155	1.235 ± 0.161	1.251 ± 0.170	0.008 ± 0.036	0.008 ± 0.039	0.007 ± 0.042
BMD total hip (g/cm^2^)	1.107 ± 0.133	1.067 ± 0.128	1.092 ± 0.130	0.008 ± 0.014	0.011 ± 0.014	0.009 ± 0.017
OPG (pg/ml)	1875 ± 509	1961 ± 600	2092 ± 650	56 ± 306^1^		- 34 ± 472^2^
RANKL (pg/ml)	0.09 ± 0.15	0.10 ± 0.27	0.05 ± 0.10	- 0.01 ± 0.10^1^		0.00 ± 0.06^2^
Serum 25(OH)D (nmol/L)	61.3 ± 20.7	58.3 ± 21.2	60.1 ± 22.3	79.9 ± 31.3^†^	41.7 ± 22.8^†^	- 2.2 ± 16.8
Serum PTH (pmol/L)	5.1 ± 1.6	5.4 ± 1.8	5.7 ± 1.7	- 0.9 ± 1.5^†^	- 0.7 ± 1.4*	- 0.2 ± 1.6
Serum calcium (mmol/L)	2.30 ± 0.11	2.32 ± 0.11	2.31 ± 0.10	- 0.01 ± 0.12	- 0.02 ± 0.12	- 0.01 ± 0.11

*P < 0.05; ^†^*P *< 0.001 versus delta values in the PP grooup

^1^N = 76; ^2^N = 81

At the end of the study serum 25(OH)D levels were 140.9 ± 34.7 nmol/L, 99.7 ± 20.3 nmol/L, and 57.9 ± 20.4 nmol/L in the DD, DP and PP groups respectively. There was a highly significant positive correlation between baseline and 12 months serum 25(OH)D in both the DD and DP groups (r = 0.40 and r = 0.69, respectively (*P *< 0.001)), and a significant negative correlation between baseline and delta serum 25(OH)D (r = - 0.57 and r = - 0.49, respectively (*P *< 0.001)). Accordingly, in those given vitamin D, those with the lowest baseline serum 25(OH)D levels had the highest increase in serum 25(OH)D, but not high enough to even out the differences seen at baseline. There was a significant decrease in the serum PTH levels in the DD and DP groups, but no significant changes in weight or serum calcium. The three groups did not differ significantly in delta values for BMD L2-L4, BMD hip, serum OPG or serum RANKL (Table 4), not even when the DD and DP groups were combined to one vitamin D group and compared with the PP group, or when only subjects with low serum 25(OH)D values (< the 25^th ^percentile (< 45.0 nmol/L)) were evaluated separately (data not shown).

One subject in the PP group had an increase of 11.3% and one subject in the DD group had a decrease of 11.5% in BMD L2-L4. Apart from these two subjects none had an increment or a decrement outside the "least significant change" (2.8 × CV) regarding the bone densitometry.

Among the 66 women in the DD group, 58 women in the DP group, and 64 women in the PP group, 29, 28 and 35 women, respectively, were postmenopausal. However, the delta values for these women did not differ significantly between the DD, DP and PP groups, being for BMD L2-L4 0.004 ± 0.03 g/cm^2^, - 0.006 ± 0.035 g/cm^2^, and - 0.006 ± 0.035 g/cm^2^, respectively; and for BMD hip 0.003 ± 0.013 g/cm^2^, 0.008 ± 0.015 g/cm^2^, and 0.003 ± 0.014 g/cm^2^, respectively.

### Adverse events

As previously described in detail [16], no serious adverse events were seen and there were no significant differences between the treatment groups regarding adverse events. Two subjects were diagnosed as having primary hyperparathyroidism during the study, and one had an increase in serum calcium to 2.62 mmol/L, and all three were excluded from the study. Four subjects had transient increases in serum calcium > 2.59 mmol/L and remained in the study.

## Discussion

In the present study we found no significant associations for serum 25(OH)D with BMD, serum OPG or serum RANKL at baseline after adjustment for confounders, nor did vitamin D supplementation for one year differ form placebo regarding change in BMD, serum OPG or serum RANKL. On the other hand, a high serum PTH level was associated with reduced BMD in the spine and the hip.

Regarding the negative association between PTH and BMD this is in line with previous publications from large cross-sectional studies [20,21], whereas the lack of association between 25(OH)D and BMD differs from the report by Bischoff-Ferrari et al. [14], and was most likely due to selection and number of subjects in our study. Apart from the expected significant increase in serum OPG with age and the higher levels in females [22], OPG or RANKL were not significantly associated with any of the other variables included in the study. In particular, there was after adjustment for confounders no significant relation between OPG and BMD, which is similar to that reported by Indridason et al. in a study including 1630 subjects [23].

In the intervention study, our vitamin D doses of 20.000 IU and 40.000 IU per week were substantially higher than those usually given in osteoporosis studies. This resulted in serum 25(OH)D levels in the high physiological range [24], but no significant change in BMD was found. However, a positive effect of vitamin D on BMD cannot be ruled out from our study as most of the subjects had normal BMD at baseline, the study only lasted 12 months, and an increase in BMD would therefore be hard to disclose. On the other hand, supplementation with vitamin D could in theory also have a negative effect on BMD as the production of 1,25(OH)_2_D from 25(OH)D is substrate dependent [25] and 1,25(OH)_2_D may induce osteoclastogenesis [5,11]. Our result is therefore of importance as it indicates that high doses of vitamin D, at least when given for a short period of time to healthy subjects, do not have serious adverse effects on bone. However, it must be emphasised that a direct and negative effect of vitamin D could be masked in our study by the concomitant fall in serum PTH, as a fall in serum PTH may have a beneficial effect on BMD [21,26].

Although 1,25(OH)_2_D has been demonstrated in in-vitro studies to increase RANKL expression [5] and reduce the OPG expression [9], supplementation with vitamin D in our study did not significantly affect their serum levels. In other studies where serum OPG and RANKL have been measured after therapy, the results have varied [27]. Thus, both serum OPG and RANKL have been found to decrease after treatment with oestrogen in postmenopausal women in one study [28], whereas serum OPG but not RANKL increased after oral contraceptives in another study [29]. No effect on OPG by bisphosphonate therapy has been reported in subjects with osteoporosis or rheumatoid arthritis [30,31], whereas a decrease in OPG after bisphosphonates has been found in subjects with Paget's disease [30]. Most likely oestrogen, as well as bisphosphonates, influence the production of OPG and RANKL as demonstrated in in-vitro studies [32,33]. Therefore, the lack of corresponding changes in serum levels indicate that the circulating levels of OPG and RANKL do not reflect the concentrations in the local tissues [27].

Our study has several limitations, and the results should be evaluated with caution. Thus, the effect of vitamin D on BMD, serum OPG and RANKL were only secondary endpoints, most of the included subjects were not vitamin D deficient, and serum 1,25(OH)_2_D was not measured. All subjects were given calcium supplementation and the results should therefore be interpreted as vitamin D plus calcium versus calcium alone. On the other hand, our study is of importance as we used high vitamin D doses resulting in serum 25(OH)D levels in the high physiological range.

## Conclusion

Supplementation with high doses of vitamin D for one year did not have a negative effect on BMD at the spine or hip in healthy overweight or obese subjects, and a relation between serum levels of vitamin D and OPG and RANKL could not be demonstrated.

However, in order to disclose a positive effect on BMD, subjects with low BMD and/or low serum 25(OH)D levels need to be studied for a longer period of time.

## Competing interests

The authors declare that they have no competing interests.

## Authors' contributions

RJ: study design, data gathering, preparation manuscript. MS: data gathering, preparation manuscript. PAT: vitamin D analyses, preparation manuscript. YF: biochemical analyses, preparation manuscript. JBH: OPG and RANKL analyses, preparation manuscript. GG: statistical analyses, preparation manuscript. All authors have read and approved the final manuscript.
